# Impact of single nucleotide polymorphism on short stature and reduced tongue pressure among community-dwelling elderly Japanese participants: a cross-sectional study

**DOI:** 10.1186/s12199-017-0668-x

**Published:** 2017-07-27

**Authors:** Yuji Shimizu, Shimpei Sato, Yuko Noguchi, Jun Koyamatsu, Hirotomo Yamanashi, Miho Higashi, Mako Nagayoshi, Koichiro Kadota, Shin-Ya Kawashiri, Yasuhiro Nagata, Noboru Takamura, Takahiro Maeda

**Affiliations:** 1 Department of Community Medicine, Nagasaki University Graduate School of Biomedical Sciences, Nagasaki-shi, Sakamoto 1-12-4, Nagasaki, 852-8523 Japan; 2 Department of Cardiovascular Disease Prevention, Osaka Center for Cancer and Cardiovascular Disease Prevention, Osaka, Japan; 3 Research and Clinical Center for Yusho and Dioxin, Kyusyu University, Fukuoka, Japan; 4 Department of Island and Community Medicine, Nagasaki University Graduate School of Biomedical Sciences, Nagasaki, Japan; 5 Department of Global Health, Medicine and Welfare, Nagasaki University Graduate School of Biomedical Sciences, Nagasaki, Japan; 6 Center for Comprehensive Community Care Education, Nagasaki University Graduate School of Biomedical Sciences, Nagasaki, Japan

**Keywords:** SNPs, rs3782886, Tongue pressure, Height

## Abstract

**Background:**

Asian-specific single nucleotide polymorphism (SNPs) (rs3782886) is reported to be associated with myocardial infarction; sarcopenia is reported to be associated with coronary subclinical atherosclerosis. On the other hand, short stature has been revealed as an independent risk factor for cardiovascular disease. However, no studies have reported on the association between sarcopenia and short stature nor on the impact of rs3782886 on this association.

**Methods:**

Since reduced maximum voluntary tongue pressure against the palate (MTP) reflects one aspect of sarcopenia, we conducted a cross-sectional study of 537 community-dwelling elderly Japanese participants aged 60–89 years who had participated in a general health checkup in 2015. Short stature was defined as values at or under the 25th percentile, and reduced MTP was defined as the lowest tertile of the study population (<158.0 cm and <26.5 kPa for men, <145.0 cm and <24.1 kPa for women).

**Results:**

Independent of classical cardiovascular risk factors, short stature was revealed to be positively associated with reduced MTP. The adjusted-odds ratio (OR) and 95% confidence interval (CI) of reduced MTP for short stature was 1.87 (1.19, 2.94). We also found that independent of known cardiovascular risk factors, with the non-minor homo of rs3782886 taken as the reference group, the adjusted OR and 95% CI for short stature and reduced MTP of the minor homo allele were 3.06 (1.23, 7.63) and 3.26 (1.33, 8.03), respectively.

**Conclusion:**

Short stature is independently associated with reduced MTP, with Asian-specific SNPs possibly playing an important role in this association.

## Background

Asian-specific single nucleotide polymorphism (SNP) rs3782886 in breast cancer suppressor protein-associated protein (BRAP) is reported to be associated with a risk of myocardial infarction [1]. Another study reported that BRAP activates inflammatory cascades and increases the risk of carotid atherosclerosis [2]. Since Japanese individuals are known to have a short stature, and short stature may constitute an inflammatory disadvantage [3] and a risk of carotid atherosclerosis [4], SNP rs3782886 may therefore be associated with short stature.

On the other hand, sarcopenia is also reported to be associated with coronary subclinical atherosclerosis in the very elderly [5], and age-associated changes to the immune system that induce part of the inflammatory reaction have been suggested to contribute to sarcopenia [6]. Furthermore, reduced maximum voluntary tongue pressure against the palate (MTP) is reported to be associated with sarcopenia [7, 8].

Short stature may therefore be associated with decreased MTP by indicating possible inflammatory disadvantage in participants with short stature [3].

Additionally, SNP rs3782886 might also be associated with reduced tongue pressure as an indicator of Asian-specific inflammatory disadvantage leading to sarcopenia in elderly Japanese participants.

On the other hand, height is regarded as a marker of childhood social and physical conditions [4, 9, 10]. Since BMI, which is reported to be associated with increased risk of disease [11], is largely influenced by current circumstances, studies limited to participants with lower BMI might allow elucidation of the influence of childhood circumstances (including genetic factors) by weakening the influence of current circumstances [9, 12].

To clarify these associations, we conducted a cross-sectional study of 537 community-dwelling elderly Japanese aged 60–89 years who had participated in general health checkup in 2015.

## Methods

### Study population

The total number of residents of Goto city aged 60–89 in 2015 (estimated by the National Institute of Population and Social Security Research in March 2013) was 16,176 [13]. Among them, 579 participants were taking an annual medical checkup, of which 33 did not agree to participate in our present study (participation rate 94.3%). Therefore, the study population comprised 546 Japanese elderly residents from the western rural communities of the Goto Islands, who undertook an annual medical checkup in 2015 as recommended by the Japanese government.

Those without MTP data (*n* = 9) were excluded, leaving 537 participants with a mean age of 73.4 years (standard deviation (SD) 7.5; range 60–89) enrolled in the study.

### Data collection and laboratory measurements

Trained interviewers obtained information on clinical characteristics. Body weight and height were measured with an automatic body composition analyzer (BF-220; Tanita, Tokyo, Japan), and body mass index (BMI; kg/m^2^) was calculated. Systolic and diastolic blood pressure were recorded at rest. Primarily fasting blood samples were collected in a siliconized tube. Triglycerides (TG) and creatinine were measured enzymatically. HDL-cholesterol (HDL) was measured using a direct method, while hemoglobin A1c (HbA1c) was measured using the latex coagulation method at SRL, Inc. (Tokyo, Japan). Genomic DNA was extracted from 2 mL of whole peripheral blood with GENE PREP STAR NA-480 (KURABO). Subject DNA was typed for SNP rs3782886 (BRAP on chromosome 12q24.12) using the HybProbe method with LightCycler 480 (Roshe). MTP was evaluated by the method proposed by Tsuga et al. using the JMS tongue pressure measurement device, Orarize (TPM-01, JMS Co., Ltd. Hiroshima, Japan) [14, 15]. This tongue pressure measuring device consists of a disposable oral probe. To measure MTP, participants were placed in a relaxed sitting position and asked to compress the disposal balloon by raising their tongue using maximum voluntary effort.

Since a previous study of community-dwelling elderly individuals reported that individuals in the lowest tertiles of tongue pressure had lower scores in the muscle function test (handgrip strength, jump height, and jump power) [8] than individuals in other tertiles, we defined reduced MTP as a tongue pressure in the lowest tertile of the study population (<26.5 kPa for men and <24.1 kPa for women). And short stature was defined as a height level at or under the 25th percentile of the study population (<158.0 cm for men and <145.0 cm for women) since these participants may have cardiovascular risk factors determined by childhood social and physical conditions (including genetic factors) [9, 12].

### Statistical analysis

Clinical characteristics of the SNP rs3782886 genotype were compared. Differences in mean ± SD values or prevalence of potential confounding factors by SNP rs3782886 genotypes were calculated. A trend test was performed with a regression model for mean values, and a logistic regression model was used for proportion.

Logistic regression models were used to calculate odds ratios (ORs) and 95% confidence intervals (CIs) to determine the influence of height and short stature on reduced MTP among sex-combined model and sex-specific models. We also used logistic regression models to calculate ORs and 95% CIs to determine the influence of SNP rs3782886 on short stature and reduced MTP in a sex-combined model and among women. And a further analysis of the total participants limited to those with a BMI < 25 kg/m^2^ was also conducted since this might elucidate the influence of childhood circumstances (including genetic factors).

Adjustments for confounding factors were made into two models. In the first model (model 1), we adjusted only for sex and age. In the second (model 2), we included other possible confounding factors, such as BMI (kg/m^2^), systolic blood pressure (mmHg), alcohol consumption [never drinker, former drinker, current drinker (<23 g/week, 23 to 45g/week, 46 to 68 g/week, ≥69 g/week)], smoking status (never smoker, former smoker, current smoker), history of stroke (yes, no), HDL-cholesterol (mg/dL), triglycerides (mg/dL), HbA1C (%), and serum creatinine (mg/dL).

All statistical analyses were performed with the SAS system for Windows (version 9.4; SAS Inc., Cary, NC). All *p* values for statistical tests were two-tailed, with values of <0.05 regarded as being statistically significant.

## Results

Of the total study population, regarding the rs3782886 genotype, 326 participants were major homo (A/A), 187 hetero (A/G), and 24 minor homo (G/G).

Characteristics of the present study population are shown in Table 1. Current drinker status and height were found to be significantly associated with genotype.

**Table 1:** Characteristics of the study population by rs3782886 genotype

	rs3782886			*p* value
	Major homo (A/A)	Hetero type (A/G)	Minor homo (G/G)	
No. of participants	326	187	24	
Men, %	35.9	43.9	25.0	0.081
Age, years	73.1 ± 7.5	73.3 ± 7.7	74.2 ± 7.6	0.609
Current drinker, %	28.5	13.4	0.0	<0.001
Current smoker, %	6.1	5.9	4.2	0.925
History of stroke	4.6	4.3	12.5	0.200
Body mass index (BMI), kg/m^2^	23.3 ± 3.2	24.1 ± 3.7	23.6 ± 3.2	0.039
Systolic blood pressure, mmHg	140 ± 18	139 ± 17	137 ± 17	0.841
Diastolic blood pressure, mmHg	80 ± 11	78 ± 12	80 ± 12	0.134
Serum HDL-cholesterol (HDL), mg/dL	59 ± 15	56 ± 14	57 ± 13	0.146
Serum triglycerides (TG), mg/dL	104 ± 61	102 ± 54	99 ± 47	0.861
Hemoglobin A1c (HbA1c), %	5.7 ± 0.5	5.7 ± 0.5	5.8 ± 0.7	0.845
Serum creatinine, mg/dL	0.75 ± 0.21	0.79 ± 0.21	0.77 ± 0.22	0.140
Height, cm	154.3 ± 8.8	154.2 ± 8.8	148.7 ± 7.6	0.011

Values: mean ± standard deviation

Table 2 shows the ORs and 95% CIs for reduced MTP in relation to height level. Independent of known cardiovascular risk factors, height was inversely associated with faster reduced MTP. The classical cardiovascular risk factor-adjusted OR and 95% CI of reduced MTP for a decrement of 1 SD (standard deviation) in height (6.55 cm for men and 5.63 cm for women) was 1.27 (1.02–1.58). We also evaluated the impact of short stature on reduced MTP and found a positive association between the two. With non-short stature as the reference group, the adjusted OR and 95% CI of reduced MTP for short stature was 1.87 (1.19, 2.94). When we conducted a sex-specific analysis, we found these associations to be essentially true both for men and women. With the highest tertiles of height taken as reference, the fully adjusted ORs and 95% CIs of reduced MTP were 1.70 (0.81, 3.55) for men and 1.95 (1.07, 3.54) for women.

**Table 2:** Odds ratios (OR) and 95% confidence intervals (CI) for reduced maximum voluntary tongue pressure against the palate (MTP) in relation to height

	Height				*p* for trend	1 SD decrement in height (6.55 cm for men and 5.63 cm for women)
	Q1 (short)	Q2	Q3	Q4 (tall)		
Total participants						
No. at risk	132	135	134	136		
No. of cases (percentage)	65 (49.2)	48 (35.6)	36 (26.9)	31 (22.8)		
Model 1	1.93 (1.08, 3.43)	1.35 (0.77, 2.37)	1.01 (0.57, 1.79)	1.00	0.012	1.24 (1.00, 1.54)
	1.69 (1.10, 2.61)	1.00			0.017	
Model 2	2.19 (1.20, 3.98)	1.35 (0.76, 2.39)	1.11 (0.61, 1.99)	1.00	0.007	1.27 (1.02, 1.58)
	1.87 (1.19, 2.94)	1.00			0.007	
Men						
No. at risk	50	52	52	51		
No. of cases (percentage)	23 (46.0)	22 (42.3)	14 (26.9)	19 (19.6)		
Model 1	1.97 (0.76, 5.15)	1.75 (0.68, 4.53)	1.09 (0.41, 2.86)	1.00	0.096	1.19 (0.84, 1.69)
	1.51 (0.75, 3.03)	1.00			0.035	
Model 2	2.14 (0.79, 5.81)	1.55 (0.58, 4.17)	1.17 (0.42, 3.21)	1.00	0.102	1.19 (0.82, 1.72)
	1.70 (0.81, 3.55)	1.00			0.162	
Women						
No. at risk	82	83	82	85		
No. of cases (percentage)	42 (51.2)	26 (31.3)	22 (26.8)	21 (24.7)		
Model 1	2.00 (0.97, 4.12)	1.14 (0.57, 2.28)	0.97 (0.48, 1.97)	1.00	0.054	1.28 (0.98, 1.68)
	1.92 (1.09, 3.36)	1.00			<0.001	
Model 2	2.16 (1.00, 4.66)	1.21 (0.59, 2.48)	1.08 (0.51, 2.26)	1.00	0.052	1.31 (0.98, 1.73)
	1.95 (1.07, 3.54)	1.00			0.029	

Model 1: adjusted only for sex and age. Model 2: further adjusted for body mass index, systolic blood pressure, alcohol consumption (never drinker, former drinker, current drinker [<23, 23–45, 46–68, and ≥69 g/week]), smoking status (never smoker, former smoker, current smoker), history of stroke, HDL-cholesterol, triglycerides, HbA1C, and serum creatinine. Reduced maximum voluntary tongue pressure against the palate is defined as the lowest tertile of the study population (<26.5 kPa for men and <24.1 kPa for women). Height values for men are <158.0 cm for Q1, 158.0–161.8 cm for Q2, 161.9–166.5 cm for Q3, and >166.5 cm for Q4 and for women are <145.0 cm for Q1, 145.0–148.9 cm for Q2, 149.0–152.7 cm for Q3, and >152.7 cm for Q4

Table 3 shows the OR and 95% CI for short stature in relation to the rs3782886 genotype. Independent of known cardiovascular risk factors, among the total participants, a positive association was found between rs3782886 minor homo and short stature. Compared with non-minor homo (A/A and A/G), the fully adjusted OR and 95% CI of short stature for minor homo (G/G) was 3.06 (1.23, 7.63) for the total participants and 1.73 (0.57, 5.24) for women only.

**Table 3:** Odds ratios (OR) and 95% confidence intervals (CI) for short stature in relation to rs3782886 genotype

	rs3782886			*p* for trend	Minor allele frequencies
	Non-minor homo		Minor homo		
	Major homo (A/A)	Hetero (A/G)	(G/G)		
Total participants					
No. at risk	326	187	24		
No. of cases (percentage)	72 (22.1)	49 (26.2)	11 (45.8)		
Model 1	1.00	1.21 (0.78, 1.89)	3.05 (1.22, 7.61)	0.040	1.44 (1.02, 2.05)
	1.00		2.85 (1.16, 6.99)	0.023	
Model 2	1.00	1.23 (0.77, 1.98)	3.35 (1.31, 8.59)	0.033	1.50 (1.03, 2.18)
	1.00		3.06 (1.23, 7.63)	0.017	
Women					
No. at risk	209	105	18		
No. of cases (percentage)	48 (23.0)	27 (25.7)	7 (38.7)		
Model 1	1.00	1.19 (0.66, 2.16)	1.94 (0.63, 5.94)	0.257	1.29 (0.83, 2.02)
	1.00		1.83 (0.61, 5.49)	0.284	
Model 2	1.00	1.22 (0.66, 2.27)	1.87 (0.60, 5.79)	0.261	1.30 (0.82, 2.06)
	1.00		1.73 (0.57, 5.24)	0.330	

Model 1: adjusted only for age (and sex for total participants). Model 2: further adjusted for body mass index, systolic blood pressure, alcohol consumption (never drinker, former drinker, current drinker [<23, 23–45, 46–68, and ≥69 g/week]), smoking status (never smoker, former smoker, current smoker), history of stroke, HDL-cholesterol, triglycerides, HbA1C, and serum creatinine. Short stature is defined as a height level at or under the 25th percentile of the study population (<158.0 cm for men and <145.0 cm for women)

Table 4 shows the OR and 95% CI for reduced MTP in relation to the rs3782886 genotype. Independent of known cardiovascular risk factors, a positive association was found between rs3782886 minor homo and reduced MTP. Compared with non-minor homo (A/A and A/G), the fully adjusted OR and 95% CI of reduced MTP for minor homo (G/G) was 3.26 (1.33, 8.03) for total participants and 3.04 (1.04, 8.85) for women only.

**Table 4:** Odds ratios (OR) and 95% confidence intervals (CI) for reduced maximum voluntary tongue pressure against the palate (MTP) in relation to rs3782886 genotype

	rs3782886			*p* for trend	Minor allele frequencies
	Non-minor homo		Minor homo		
	Major homo (A/A)	Hetero (A/G)	(G/G)		
Total participants					
No. at risk	326	187	24		
No. of cases (percentage)	112 (34.4)	54 (28.9)	14 (58.3)		
Model 1	1.00	0.72 (0.48, 1.09)	2.66 (1.10, 6.42)	0.805	1.04 (0.76, 1.43)
	1.00		2.97 (1.25, 7.09)	0.014	
Model 2	1.00	0.76 (0.49, 1.17)	2.92 (1.17, 7.29)	0.566	1.11 (0.79, 1.55)
	1.00		3.26 (1.33, 8.03)	0.010	
Women					
No. at risk	209	105	18		
No. of cases (percentage)	65 (31.1)	35 (33.3)	11 (61.1)		
Model 1	1.00	1.12 (0.67, 1.87)	3.37 (1.21, 9.42)	0.067	1.44 (0.98, 2.12)
	1.00		3.25 (1.18, 8.93)	0.022	
Model 2	1.00	1.14 (0.66, 1.95)	3.18 (1.07, 9.43)	0.088	1.43 (0.95, 2.15)
	1.00		3.04 (1.04, 8.85)	0.042	

Model 1: adjusted only for sex and age. Model 2: further adjusted for body mass index, systolic blood pressure, alcohol consumption (never drinker, former drinker, current drinker [<23, 23–45, 46–68, and ≥69 g/week]), smoking status (never smoker, former smoker, current smoker), history of stroke, HDL-cholesterol, triglycerides, HbA1C, and serum creatinine. Reduced maximum voluntary tongue pressure against the palate is defined as the lowest tertile of the study population (<26.5 kPa for men and <24.1 kPa for women)

Table 5 shows the OR and 95% CI for short stature and reduced MTP in relation to the rs3782886 genotype among participants with a BMI < 25 kg/m^2^. Compared with the analysis for all participants (including both BMI < 25 kg/m^2^ and BMI ≥ 25 kg/m^2^), the associations of short stature and reduced MTP in relation to rs3782886 were slightly stronger. Compared with non-minor homo (A/A and A/G), the fully adjusted ORs and 95% CIs of short stature and reduced MTP for minor homo (G/G) were 4.21 (1.36, 12.98) and 4.31 (1.41, 13.11), respectively.

**Table 5:** Odds ratios (OR) and 95% confidence intervals (CI) for short stature and reduced maximum voluntary tongue pressure against the palate (MTP) in relation to the rs3782886 genotype among participants with BMI < 25 kg/m^2^

	rs3782886			*p* for trend	Minor allele frequencies
	Non-minor homo		Minor homo		
	Major homo (A/A)	Hetero (A/G)	(G/G)		
Short stature					
Total participants (BMI < 25 kg/m^2^)					
No. at risk	232	123	17		
No. of cases (percentage)	56 (24.1)	30 (24.4)	9 (52.9)		
Model 1	1.00	0.96 (0.55, 1.68)	4.18 (1.37, 12.75)	0.127	1.39 (0.91, 2.13)
	1.00		4.23 (1.41, 12.72)	0.010	
Model 2	1.00	1.03 (0.57, 1.86)	4.25 (1.35, 13.39)	0.092	1.47 (0.94, 2.31)
	1.00		4.21 (1.36, 12.98)	0.012	
Reduced maximum voluntary tongue pressure against the palate (MTP)					
Total participants (BMI < 25 kg/m^2^)					
No. at risk	232	123	17		
No. of cases (percentage)	82 (35.3)	44 (35.8)	11 (64.7)		
Model 1	1.00	1.00 (0.62, 1.61)	3.56 (1.21, 10.53)	0.149	1.32 (0.91, 1.92)
	1.00		3.57 (1.22, 10.43)	0.020	
Model 2	1.00	1.11 (0.66, 1.86)	4.49 (1.45, 13.92)	0.054	1.49 (0.99, 2.24)
	1.00		4.31 (1.41, 13.11)	0.010	

Model 1: adjusted only for sex and age. Model 2: further adjusted for body mass index, systolic blood pressure, alcohol consumption (never drinker, former drinker, current drinker [<23, 23–45, 46–68, and ≥69 g/week]), smoking status (never smoker, former smoker, current smoker), history of stroke, HDL-cholesterol, triglycerides, HbA1C, and serum creatinine. Short stature is defined as a height level at or under the 25th percentile of the study population (<158.0cm for men and <145.0 cm for women). Reduced maximum voluntary tongue pressure against the palate is defined as the lowest tertile of the study population (<26.5 kPa for men and <24.1 kPa for women)

## Discussion

Our present study revealed height to be inversely associated with reduced MTP among community-dwelling elderly Japanese participants. Additionally, a significant association was seen between Asian-specific SNPs and short stature and reduced MTP. These results indicate that SNPs play an important role, at least in part, on the association between height and reduced MTP among elderly Japanese participants.

Our additional analysis which was limited to participants with a BMI < 25 kg/m^2^ showed slightly stronger associations with regard to short stature and reduced MTP in relation to SNP rs3782886. Since an analysis limited to participants with a lower BMI might elucidate the influence of childhood circumstances (including genetic factors) [9, 12], the additional analysis also supports the present conclusion that a genetic factor may influence the association between height and reduced MTP.

The possible mechanism underlying the positive association between short stature and reduced MTP and the role of SNP rs3782886 in this association involves low-grade inflammation. SNP rs3782886 is known to be located on the BRAP gene on chromosome 12q24, and higher expression of BRAP with the minor allele (G allele) is associated with increased risk of atherosclerosis through enhancing the degree of inflammation via activation of NF-κB protein [2, 16]. In addition, we reported in a previous study that short stature constitutes an inflammatory disadvantage among middle-aged Japanese men [3] and that height is inversely associated with carotid atherosclerosis in overweight men [4]. In our present study, we found that SNP rs3782886 with the minor homo (G/G) allele is positively associated with short stature. Therefore, short stature may result in an inflammatory disadvantage that relates to SNP rs3782886 with the minor homo (G/G) genotype.

On the other hand, decreased tongue pressure is associated with sarcopenia [7, 8], and sarcopenia is associated with coronary subclinical atherosclerosis in the very elderly [5]. Since atherosclerosis, which is one of the major risk factors for coronary artery disease, is known as an inflammatory condition [17], such a condition may form a complicated network, resulting in the present study’s observations. A previous study that reported the unfavorable effects of systematic inflammation on muscle strength in the elderly also supports this mechanism [18]. Further investigations are necessary to clarify the network involved.

Another possible mechanism at play is lower skeletal muscle capillarization resulting in the positive association between short stature and reduced MTP. Previously, we reported height to be an indicator of vascular maintenance capacity in older men [19]; sarcopenia is also reported to be associated with lower skeletal muscle capillarization [20]. Lower skeletal muscle capillarization caused by lower vascular maintenance capacity, which is associated with short stature, might result in reduced MTP.

**Fig. 1:**
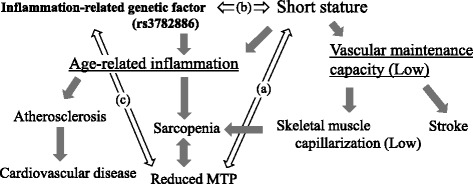
The possible mechanism underlying the positive association between short stature and reduced MTP and the role of SNP rs3782886. In our present study, **a** short stature was positively associated with reduced MTP, **b** rs3782886 minor homo was positively associated with short stature, and **c** rs3782886 minor homo was positively associated reduced MTP

A summary of the possible mechanism underlying the results of our present study is shown in Fig. 1. Age-related low-grade inflammation and low vascular maintenance capacity should take important roles in progression of sarcopenia.

Our present study is the first to report the impact of height on tongue pressure, as well as the first to reveal a significant association between SNP rs3782886 and short stature and reduced MTP. Since SNP rs3782886 is an inflammation-related genetic factor [1, 2], our present results represent an efficient tool to clarify the genetic influence of age-related inflammation on reduced MTP.

Potential limitations of this study warrant consideration. Although SNP rs3782886 is significantly associated with short stature and reduced MTP, no data was available with regard to the evaluation of endothelial function. Further analyses that include endothelial function-related data such as flow-mediated dilation (FMD) will be necessary. Since only four cases of short stature and three cases of reduced MTP among men with the minor homo allele (*n* = 6) were available, a male-specific analysis was not able to be carried out. Further investigation with a larger study population will be necessary. And since our present study population targeted elderly participants, height data may be confounded by age-related physical changes such as kyphosis and compression fracture of the spine, although we found no significant differences between age and SNP genotypes in the present study population. Finally, even though a positive association between short stature and reduced MTP was revealed, causal relationships were not able to be established since this was a cross-sectional study.

## Conclusion

In conclusion, height is inversely associated with reduced MTP among community-dwelling elderly Japanese participants. Additionally, Asian-specific SNPs were also found to be significantly associated with short stature and reduced MTP. These results indicate that genetic factors may play an important role, at least in part, on the association between height and reduced MTP among elderly Japanese participants.

## Abbreviations

BMI: Body mass index
BRAP: Breast cancer suppressor protein-associated protein
FMD: Flow-mediated dilation
HbA1c: Hemoglobin A1c
HDL: High-density lipoprotein-cholesterol
MTP: Maximum voluntary tongue pressure against the palate
OR: Odds ratio
SD: Standard deviation
SNP: Single nucleotide polymorphism
TG: Triglycerides

## References

[1] Ozaki K, Sato H, Inoue K, Tsunoda T, Sakata Y, Mizuno H (2009). SNPs in BRAP associated with risk of myocardial infarction in Asian populations. Nat Genet.

[2] Liao YC, Wang YS, Guo YC, Ozaki K, Tanaka T, Lin HF (2011). BRAP activates inflammatory cascades and increases the risk for carotid atherosclerosis. Mol Med.

[3] Shimizu Y, Yoshimine H, Nagayoshi M, Kadota K, Takahashi K, Izumino K (2016). Short stature is an inflammatory disadvantage among middle-aged Japanese men. Environ Health Prev Med.

[4] Shimizu Y, Nakazato M, Sekita T, Kadota K, Arima K, Yamasaki H (2013). Relationship between adult height and body weight and risk of carotid atherosclerosis assessed in terms of carotid intima-media thickness: the Nagasaki Islands study. J Physiol Anthropol.

[5] Campos AM, Moura FA, Santos SN, Freitas WM, Sposito AC, Brasilia Study on Healthy Aging and Brasilia Heart Study (2017). Sarcopenia, but not excess weight or increased caloric intake, is associated with coronary subclinical atherosclerosis in the very elderly. Atherosclerosis.

[6] Wilson D, Jackson T, Sapey E, Lord JM (2017). Frailty and sarcopenia: the potential role of an aged immune system. Ageing Res Rev.

[7] Maeda K, Akagi J (2015). Decreased tongue pressure is associated with sarcopenia and sarcopenic dysphagia in the elderly. Dysphagia.

[8] Buehring B, Hind J, Fidler E, Krueger D, Binkley N, Robbins J (2013). Tongue strength is associated with jumping mechanography performance and handgrip strength but not with classic functional tests in older adults. J Am Geriatr Soc.

[9] Shimizu Y, Imano H, Ohira T, Kitamura A, Kiyama M, Okada T (2014). CIRCS Investigators. Adult height and body mass index in relation to risk of total stroke and its subtypes: the circulatory risk in communities study. J Stroke Cerebrovasc Dis.

[10] Hozawa A, Murakami Y, Okamura T, Kadowaki T, Nakamura K, Hayakawa T (2007). NIPPON DATA80 Research Group. Relation of adult height with stroke mortality in Japan: NIPPON DATA80. Stroke.

[11] WHO Expert Consultation. Appropriate body-mass index for Asian populations and its implications for policy and intervention strategies. Lancet. 2004;363:157–63. 10.1016/S0140-6736(03)15268-3 14726171

[12] Shimizu Y, Yoshimine H, Nagayoshi M, Kadota K, Takahashi K, Izumino K (2016). Height correlates with dyslipidemia in non-overweight middle-aged Japanese men. J Physiol Anthropol..

[13] National Institute of Population and Social Security Research [Home page on the Internet]. [Cited July 11, 2017] Available from: http://www.ipss.go.jp/pp-shicyoson/j/shicyoson13/3kekka/Municipalities.asp.

[14] Tsuga K, Maruyama M, Yoshikawa M, Yoshida M, Akagawa Y (2011). Manometric evaluation of oral function with a hand-held balloon probe. J Oral Rehabil.

[15] Utanohara Y, Hayashi R, Yoshikawa M, Yoshida M, Tsuga K, Akagawa Y (2008). Standard values of maximum tongue pressure taken using newly developed disposable tongue pressure measurement device. Dysphagia..

[16] Karin M, Delhase M (2000). The I kappa B kinase (IKK) and NF-kappa B: key elements of proinflammatory signalling. Semin Immunol.

[17] Hansson GK (2005). Inflammation, atherosclerosis, and coronary artery disease. N Engl J Med.

[18] Degens H (2010). The role of systemic inflammation in age-related muscle weakness and wasting. Scand J Med Sci Sports.

[19] Shimizu Y, Sato S, Koyamatsu J, Yamanashi H, Nagayoshi M, Kadota K, et al. Height is an indicator of vascular maintenance capacity in older man. Geriatr Gerontol Int. 2016 [Epub ahead of print] 10.1111/ggi.12876 27562673

[20] Prior SJ, Ryan AS, Blumenthal JB, Watson JM, Katzel LI, Goldberg AP (2016). Sarcopenia is associated with lower skeletal muscle capillarization and exercise capacity in older adults. J Gerontol A Biol Sci Med Sci.

